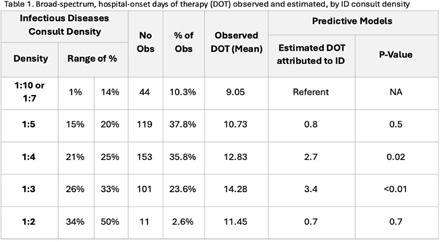# The Impact of Infectious Disease Consult on Hospitalist Prescribing of Broad-Spectrum Antibiotics

**DOI:** 10.1017/ash.2025.215

**Published:** 2025-09-24

**Authors:** Lucy Witt, Radhika Prakash Asrani, Hyun Bin Kim, Ashley Jones, Kristen Paciullo, Hasan Shabbir, Sujit Suchindran, Jesse T. Jacob, Chad Robichaux, Scott Fridkin

**Affiliations:** 1 Emory University School of Medicine; 2 Emory Healthcare

## Abstract

**Introduction:** Within our healthcare system, hospitalists receive feedback on antibiotic prescribing via an observed-to-expected ratio (OER) calculated by days of therapy (DOT) for CDC defined broad-spectrum, hospital-onset (BSHO) antibiotics and adjusted for patient characteristics and billing. In this sub-analysis, we quantify the impact of infectious disease (ID) consultations on OER. **Methods:** For each two-month period in five hospitals, encounters were assigned to each hospitalist if they billed for ≥1 day of care. The encounter was considered to involve an ID consult if an ID provider billed during the encounter. Percent of encounters with ID consultation (density) was calculated and stratum defined by gross ratios (e.g., 1 in 3 or 1 in 4 patients). We assessed whether consult density varied overtime, by facility, or by DOT. We assessed the effect of consult density on antibiotic DOT using established linear mixed effects model with random intercepts for both provider and facility (nested) and adjusted for patient characteristics and billing. Distribution of OERs were compared among strata to evaluate how ID consult changes OERs. **Results:** Between January and June 2023, 154 unique providers collectively received 458 bi-monthly OERs reflecting their care for 53,815 unique patients. Overall, 21% of hospital medicine patients were evaluated by an ID consultant during inpatient stay; median consultation density varied among providers by facility (19%-26%, Figure 1). Multivariate models (accounting for sepsis, UTI, renal disease) estimated significantly increased DOT for hospitalists having ~1:3 (+3.4 DOT, 95% CI 0.9 – 5.9) or 1:4 (+2.7 DOT, 95% CI 0.4-5.0) patients with ID consults compared to hospitalists with fewer than ~1:7 with an ID consult; however the effect was not significant in other strata and not linear (Table 1). Calculating the distribution of OERs both before and after adjusting for consult density resulted in small changes in OERs (Figure 1b). **Discussion:** The frequency of ID consults affected hospitalists’ BSHO-DOT in a non-linear fashion. Impact of ID consultation on prescribing metrics should be considered in building credibility of stewardship prescribing performance metrics.

**Figure:**
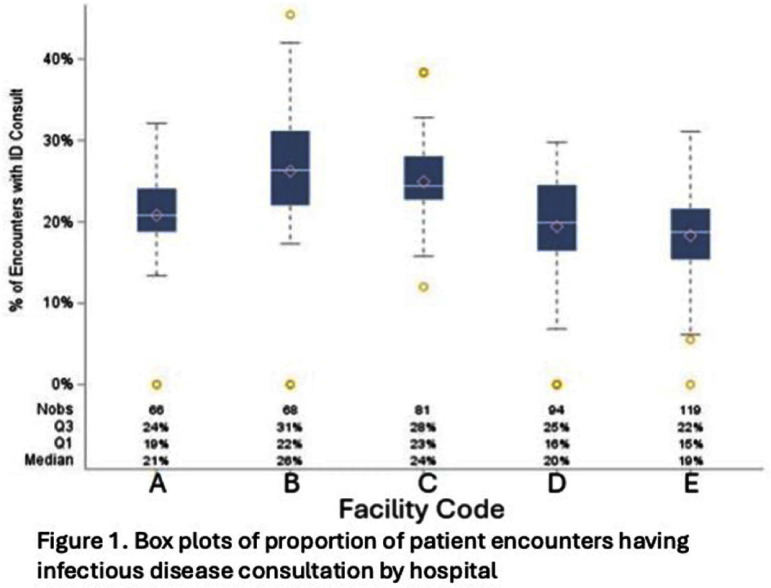

**Figure:**
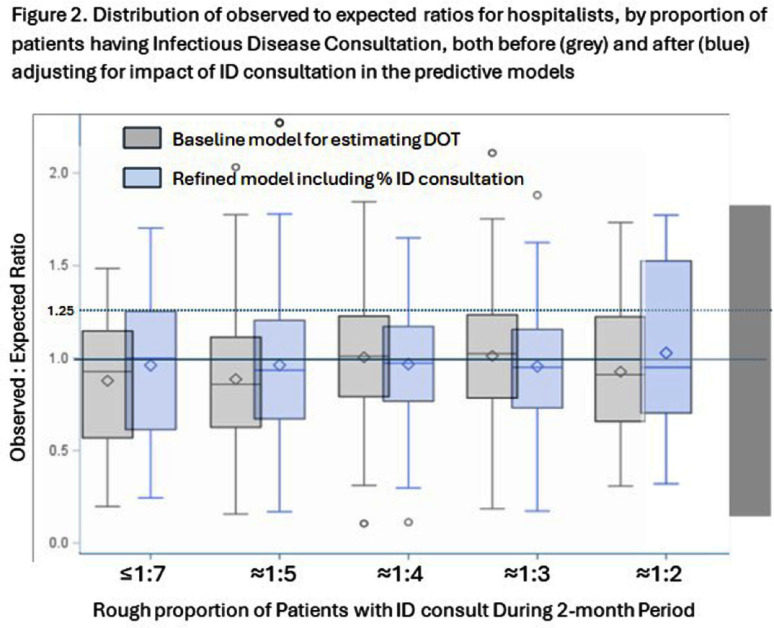

**Figure:**